# Evolution and challenges of physician-led home visits in Japan

**DOI:** 10.1093/intqhc/mzaf102

**Published:** 2025-09-23

**Authors:** Kyohei Daigo, Shun Kohsaka, Yasuyuki Shiraishi, Masaki Ieda

**Affiliations:** Department of Cardiology, Keio University School of Medicine, 35 Shinanomachi Shinjuku-ku, Tokyo 160-8582, Japan; Department of Cardiology, Keio University School of Medicine, 35 Shinanomachi Shinjuku-ku, Tokyo 160-8582, Japan; Department of Cardiology, Keio University School of Medicine, 35 Shinanomachi Shinjuku-ku, Tokyo 160-8582, Japan; Department of Cardiology, Keio University School of Medicine, 35 Shinanomachi Shinjuku-ku, Tokyo 160-8582, Japan

## Home-based healthcare in Japan

Worldwide, rapidly ageing populations and rising multimorbidity have heightened interest in physician-led home visits (PLHVs), and Japan is no exception [1]. In response to the escalating healthcare demands associated with aging, multimorbidity, and the corresponding strain on tertiary hospitals, Japanese policymakers have progressively transitioned patient care from tertiary hospitals to community-based settings. This transition began in 2006 with the introduction of reimbursement incentives for ‘home care support clinics’ by the Ministry of Health, Labor, and Welfare (MHLW) [2].

Driven largely by favorable financial incentives, these efforts further expanded in 2012 with the establishment of the ‘enhanced home care support clinic’ system. To qualify for enhanced status, clinics must meet substantially higher staffing and performance standards; including 24‑hour availability, employment of at least three full‑time physicians, and documentation of at least ten physician-led emergency home visits and four end-of-life cares annually. Despite these requirements, national statistics demonstrated rapid growth of PHLV over the past decade (Fig. 1A and B). Enhanced home care support clinics now provide more frequent emergency home visits and are associated with a lower hospital admission rate (21.5% vs. 32.2%) and deliver more end-of-life care at home, resulting in a higher proportion of home deaths compared to conventional home care clinics (80.9% vs. 64.9%) [3]. Given that home has ranked as the most preferred place of death among older adults, the expansion of PLHVs aligns closely with the principles of patient-centered care [4].

**Figure 1:**
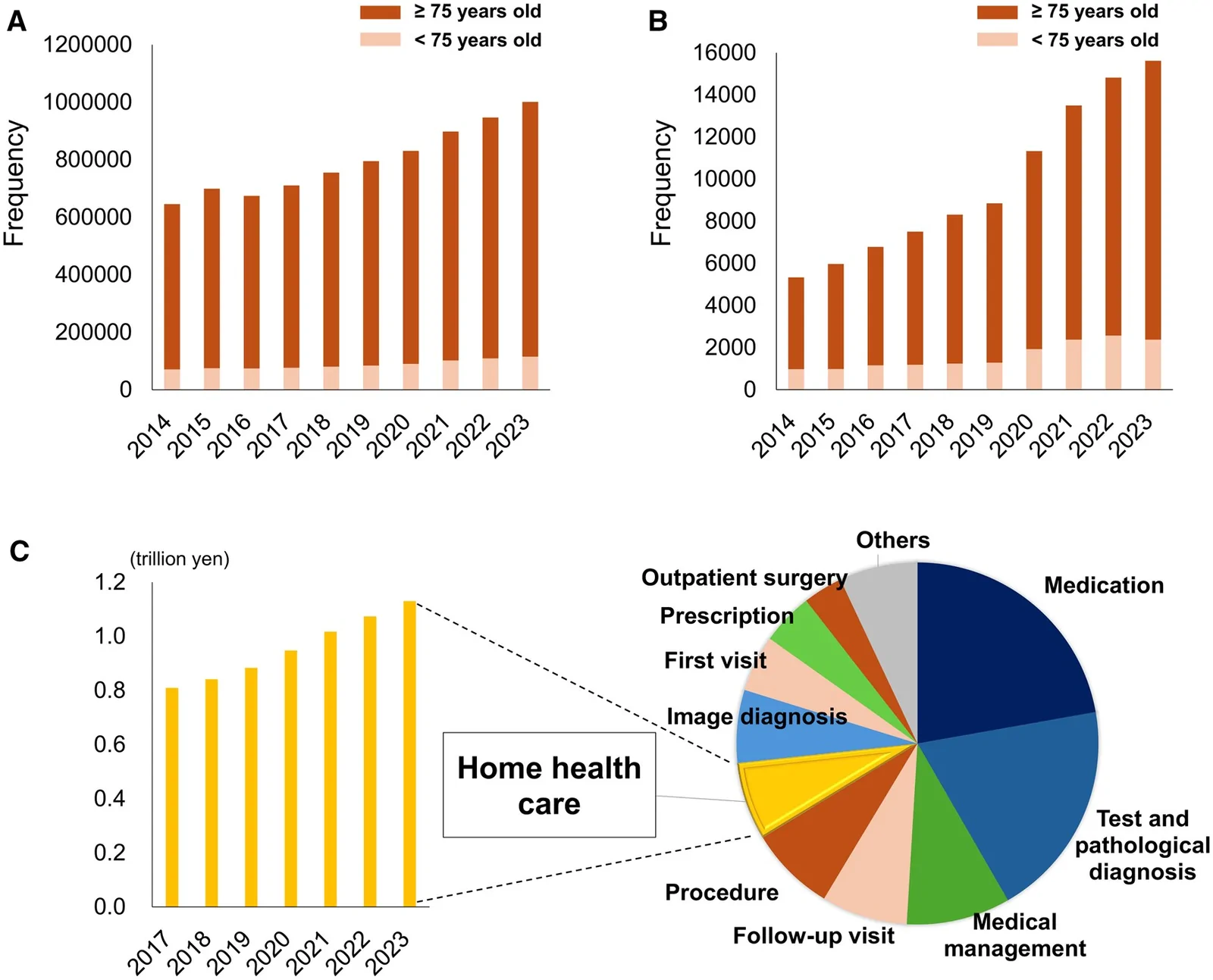
Time trends in monthly counts of (A) physician-led home visits and (B) death certifications at home during the provision of home-based care, as estimated from electronic health insurance claims data. (C) Time trends in home healthcare expenditures and the proportion of the costs relative to total annual outpatient expenditures in 2023. All data were obtained from the portal site of the Japanese Government Statistics (https://www.e-stat.go.jp/).

## Global benefits of home-based healthcare

The clinical benefits of home-based healthcare have been well documented in international studies. In Canada, a large cohort study of 49,613 older adults found that patients receiving more frequent home visits by primary care physicians had markedly lower rates of emergency department use and hospital admissions [5]. Similarly, in the United States, a randomized clinical trial of home-based primary care for homebound older adults showed that patients receiving PLHVs reported higher satisfaction with care and experienced a 17.9% reduction in hospitalization rates compared to those receiving office-based primary care [6]. These findings, along with Japan’s recent policy momentum supporting PLHV adoption, underscore the potential of PLHV to enhance patient outcomes while alleviating pressure on acute care systems.

## Financial challenges

Although the expansion of PLHVs has been expected to contribute to community-based, patient-centered care models, financial sustainability remains a concern. At present, the reimbursement rate for PLHV significantly exceeds standard outpatient care: a standard outpatient visit is reimbursed at 750 JPY per visit, while PLHV is reimbursed at 8880 JPY per visit (typically conducted twice a month), representing a more than ten-fold difference. As a result, expenditures on home healthcare have escalated sharply to approximately one trillion yen annually, accounting for a substantial 7% of all outpatient care costs (Fig. 1C).

Recognizing growing fiscal pressures, in 2024, MHLW reduced reimbursement rates for emergency and after-hours home visits to curb rising costs and encourage clinically appropriate, timely care [7]. The policy aims to prioritize visits delivered by the patient’s attending physician and to discourage unnecessary emergency call-outs. This marks a shift from reimbursement-driven expansion toward a more sustainable quality-focused model.

## Reassessment of physician-led home visit models and future directions

Japan stands at a pivotal moment in redefining home-based healthcare. Sustaining PLHV will require careful patient selection, thoughtful reimbursement reform, and technological integration. These challenges are increasingly shared across aging societies worldwide. Currently, PLHVs in Japan serve patients from routine medication to complex ventilator-dependent care, yet the basic reimbursement structure remains uniform regardless of patient complexity. This misalignment underscores a broader lesson: reimbursement models must reflect patient heterogeneity if home-based care is to remain viable.

As demographic and resources pressures tighten globally, policymakers must re-evaluate and adapt PLHVs to ensure alignment with patient needs and value of care. International experience offers instructive examples. In the United Kingdom, coding-based algorithms have been applied to refine patient selection and promote equitable allocation of resources [8]. In France, physician-operated remote monitoring programs have helped mitigate workforce burden while preserving quality, and the UK has expanded this approach into virtual wards [9,10]. For patients with heart failure, such programs have reduced readmissions and healthcare costs compared with usual care. These developments highlight the global relevance of Japan’s PLHV experience. By combining technology with personalized physician involvement, home-based care models can extend reach, enable timely interventions, prioritize urgent in-person visits, and deliver sustainable, individualized, preference-concordant care.

## Conclusions

PLHVs represent an evidence-based approach to delivering high-quality care for older adults and have expanded in Japan under reimbursement-driven incentives. However, long-term sustainability is threatened by rising costs, heterogeneous care needs, and limited alignment between service intensity and patient vulnerability. Addressing these challenges will require more targeted patient selection, reimbursement reform based on care complexity, and integration of remote monitoring technologies. A shift toward a more focused, value-based, and technology-enabled model is essential to sustaining the benefits of home-based care in an aging society.

## Data Availability

The datasets were derived from sources in the public domain: e-Stat, Portal Site of Official Statistics of Japan, available at https://www.e-stat.go.jp/jp.
